# Overcoming Electronic Medical Records Adoption Challenges in Saudi Arabia

**DOI:** 10.7759/cureus.53827

**Published:** 2024-02-08

**Authors:** Anas Alhur

**Affiliations:** 1 Health Informatics, University of Hail College of Public Health and Health Informatics, Hail, SAU

**Keywords:** patient data privacy and security, digital health transformation, vision 2030, saudi arabia, healthcare information technology, electronic medical records

## Abstract

In the digital era, the seamless integration of electronic medical records (EMR) stands as a pivotal milestone in transforming healthcare delivery, with Saudi Arabia at the forefront of this revolution in the Middle East. This literature review comprehensively explores the challenges and opportunities associated with adopting EMR in the Kingdom of Saudi Arabia (KSA) in alignment with the nation's Vision 2030 healthcare objectives. The review synthesizes research from various scholarly sources, utilizing databases such as PubMed, Scopus, Google Scholar, and regional databases, and focuses on literature published between 2010 and 2023. Our methodology included a strategic combination of keywords and a stringent selection criterion to ensure a focus on relevant EMR adoption studies within KSA. The review addresses key aspects of EMR adoption, including technical challenges, financial constraints, human factors, cultural and organizational barriers, privacy and security concerns, and policy and regulatory challenges. It also explores the integration of EMR with other digital health initiatives like telehealth, personal health records, and community pharmacy services. The findings reveal a complex interplay of factors influencing EMR adoption, highlighting the need for comprehensive strategies that address technical, financial, cultural, and policy-related barriers. The review concludes that while significant challenges exist, strategic approaches and solutions tailored to the specific context of Saudi Arabia can effectively facilitate EMR integration, thereby enhancing healthcare quality and efficiency in line with the nation's Vision 2030 goals.

## Introduction and background

In the era of digital transformation, the shift from traditional paper-based health records to electronic medical records (EMR) represents a significant evolution in healthcare delivery. EMRs, which digitize patients' medical histories, enable swift access to patient data, enhancing care coordination and efficiency - starkly contrasting the limited accessibility of paper records. This digital transition is a key component of healthcare modernization worldwide, with the Kingdom of Saudi Arabia (KSA) leading this revolution in the Middle East as part of its Vision 2030 healthcare goals [1].

The implementation of EMR in KSA is crucial for realizing the Vision 2030 objectives, reflecting the nation's dedication to utilizing technology to improve healthcare. However, this transformation faces unique challenges and opportunities, influenced by the country's specific healthcare infrastructure, cultural context, and technological landscape [1].

Key to this digital healthcare transformation are initiatives like telemedicine, health information exchange, and mobile health platforms, which are integral to improving patient care, efficiency, and cost savings. A study done by Alzghaibi et al. in 2023 emphasizes the importance of electronic health records (EHRs) in enhancing patient outcomes, healthcare efficiency, and reducing costs, highlighting the pivotal role of digital records in healthcare improvement [2].

The perspective of the Saudi public towards extended community pharmacy services (ECPSs), as explored by Alghamdi et al. (2023), provides insight into the broader context of healthcare digitalization, indicating a generally positive attitude but also significant barriers such as privacy concerns and communication challenges with healthcare professionals [3].

The role of personal health records (PHR), an extension of EMR, is vital in understanding the broader landscape of EMR adoption. Research by Alanazi et al. (2023) indicates a high intention among Saudi patients to use PHR, albeit with ongoing concerns about privacy and information accuracy [4]. This is in line with findings from a study by Alhur in 2022, which further corroborates the observed trends in patient attitudes towards PHR [5].

Furthermore, integrating telehealth services with EMR systems is a crucial aspect of digital healthcare. Studies indicate a significant willingness and positive attitudes towards telehealth in Saudi Arabia, suggesting a growing acceptance of this technology in the healthcare sector [6]. Research by Wali et al. (2023) in Jeddah, Saudi Arabia, assessing clinicians' perceptions and satisfaction with telehealth integrated with EMR, found high satisfaction with virtual visits, though barriers such as patients' limited technical knowledge and access to technology persist [7].

Research on digital health literacy and web-based health information-seeking behaviors in the Saudi population by Alhur et al. (2023) highlights the importance of digital literacy in healthcare, pointing to the need for improved digital health literacy to facilitate the effective adoption of EMR systems [8].

The COVID-19 pandemic has underscored the role of public health informatics in managing health crises. Studies on the COVID-19 dashboard in KSA illustrate the critical function of digital platforms in disseminating and visualizing health information, which is crucial for successfully implementing EMR systems [9,10].

Lastly, integrating artificial intelligence (AI) in healthcare, especially in mental health services, marks a significant aspect of digital transformation. Research exploring Saudi individuals' knowledge and attitudes towards AI-integrated telemental health services reveals potential challenges and opportunities in EMR adoption related to AI [11].

This paper aims to conduct an in-depth literature review focusing on the challenges associated with the adoption of EMR in KSA. By examining technical, financial, human, cultural, and policy-related challenges, along with privacy and security issues, this study seeks to provide a comprehensive understanding of the obstacles hindering EMR adoption in the Saudi healthcare context. The ultimate goal is to offer insights that can aid policymakers, healthcare professionals, and stakeholders in developing effective strategies for successful EMR integration, advancing KSA's healthcare delivery system in alignment with Vision 2030.

## Review

Technical challenges

Recent studies have provided valuable insights into the technical challenges of EMR adoption in Saudi Arabia. Almazroi et al. (2022) conducted a pivotal empirical study that examined the factors influencing the adoption of eHealth services, including EMR. Their research expanded the technology acceptance model to include trust, privacy, and system quality factors, emphasizing the significant impact of perceived usefulness and privacy on eHealth adoption. This study is particularly instructive for healthcare policymakers in Saudi Arabia, offering guidance for future efforts to maximize the benefits of eHealth services [12].

In a related context, Alateyah's 2014 research focused on the development and widespread adoption of e-government services in Saudi Arabia, closely aligning with the challenges faced in eHealth services like EMR. The study identified key factors such as trust, privacy, security, and cultural aspects, which are also crucial in the context of EMR adoption. These findings provide an integrated model that could help the Saudi government address public concerns about using online services, including EMR systems [13].

Additionally, Alssbaiheen and Love's study done in 2015 on the adoption of m-government services in Saudi Arabia, particularly by the Ministry of Higher Education and Technical and Vocational Training Corporation, highlighted similar challenges. The research revealed barriers such as inadequate infrastructure and limited understanding among students, paralleling the obstacles encountered in EMR implementation [14].

Furthermore, Alsharif's review in 2020 of Saudi Arabia's National eHealth Strategy proposed an integrated eHealth framework aimed at effectively managing healthcare operations during pandemics. The study pointed out the perceived shortcomings of the current eHealth framework and underscored the need for additional components focusing on information management and stakeholder engagement, which are pertinent to overcoming EMR adoption challenges [15].

Financial constraints

Recent studies have broadened the understanding of the financial challenges associated with the adoption of EMR in Saudi Arabia beyond the insights provided by AlGhamdi et al. (2015) and Alqahtani et al. (2015)[16,17]. A notable study by a group of researchers in 2023 explores the economic impact of electronic attendance systems in the healthcare sector, highlighting parallels to EMR implementation. This research emphasizes the benefits of digital integration in healthcare, such as cost reductions and efficient resource management, which are also crucial for EMR systems [18].

Aljohani's (2018) investigation into the adoption of health information technology (HIT) related EHRs/EMRs offers a comparative analysis between private and public hospitals in Saudi Arabia. The study underscores the potential of EHRs/EMRs to reduce medical costs while also addressing implementation challenges, particularly those related to interoperability. These findings are significant for EMR adoption, suggesting the necessity of a unified health information system to mitigate such challenges [19].

Furthermore, Aleid et al. (2023) conducted a study to identify barriers to accessing neurosurgical services in Saudi Arabia, with a focus on financial constraints and prolonged appointment waiting times. This research provides insights into the financial barriers within healthcare services, which can be extrapolated to the challenges faced in EMR adoption, especially in terms of cost considerations and resource allocation [20].

Additionally, Al-Baity's comprehensive review (2023) of the AI revolution in digital finance in Saudi Arabia offers valuable insights for the healthcare sector. The study proposes a framework for AI's development and integration into the financial sector, highlighting the potential for AI to enhance financial management in healthcare, including in the context of EMR adoption. This perspective underscores the role of AI in achieving cost savings and efficiency improvements in healthcare financial management [21].

Human factors and resistance to change

Recent research has illuminated the complex human factors and resistance to change in the adoption of EMR in Saudi Arabia. A notable study by Alhur (2023) investigated nurses' perceptions of EMR, focusing on its usefulness and ease of use. The findings highlighted a strong correlation between these factors and the acceptance of EMR, underscoring the importance of user-friendly systems in facilitating adoption among healthcare professionals [1,12].

Similarly, Hasanain, Vallmuur, and Clark (2015) explored healthcare professionals' knowledge and preferences regarding EMR systems in Saudi public hospitals. Their research revealed a significant correlation between English literacy, education levels, and EMR literacy, suggesting that enhancing computer literacy could increase staff preferences for using EMR systems. This study points to the need for comprehensive training and educational programs to boost EMR acceptance [22].

In the context of the COVID-19 pandemic, Alzahrani et al. (2022) examined the usability of telehealth among healthcare professionals in Saudi Arabia. The study found that increased usage of telehealth was positively associated with usability scores, indicating the necessity for additional training in telehealth to improve acceptance of digital health technologies, including EMR [23].

Furthermore, Abdulaziz et al. (2023) reflected on the rapid adoption of new technologies in the Saudi healthcare system during the COVID-19 pandemic. Their study emphasized the need for continuous assessment and education about new healthcare delivery methods, which is crucial for the successful adoption of EMR [24].

Collectively, these studies highlight the significance of understanding and addressing human factors and resistance to change in EMR adoption in Saudi Arabia. They underscore the need for user-friendly systems, comprehensive training, and adaptability to new technologies among healthcare professionals.

Cultural and organizational barriers

In examining the cultural and organizational hurdles to EMR adoption in Saudi Arabia, it is imperative to consider the unique societal norms and values that shape the healthcare landscape. A 2022 study on gender integration within traditionally male-dominated workplaces sheds light on the broader cultural dynamics at play, revealing how societal perceptions and managerial attitudes towards gender roles influence organizational change, including technology adoption [25]. These insights underscore the need for EMR implementation strategies that are sensitive to gender dynamics and the broader cultural context.

Further investigation into telemedicine services by Baradwan and Al-Hanawi (2023) highlights resistance from both patients and physicians, pointing to deeper cultural apprehensions toward digital healthcare modalities. Such resistance mirrors the challenges encountered in EMR adoption, suggesting that successful implementation necessitates a nuanced understanding of cultural attitudes toward healthcare technology [26].

Research on the adoption of e-government services illuminates the pivotal role of cultural influences in shaping employee attitudes toward technological advancements [27]. These findings are directly applicable to EMR adoption, indicating that fostering a cultural shift within healthcare organizations is essential for embracing digital records.

Lastly, Alateeg and Alhammadi's (2023) study on e-commerce adoption among traditional retailers provides valuable parallels, particularly in understanding how perceived utility, ease of use, and cost concerns are influenced by cultural and organizational contexts [27]. These factors are equally critical in EMR adoption, emphasizing the importance of culturally attuned change management strategies.

In light of these considerations, our analysis extends to explore how specific cultural elements, such as attitudes towards gender roles, technological apprehension, and organizational resistance, distinctly influence EMR adoption in Saudi Arabia. By integrating these cultural insights, the paper aims to propose EMR implementation strategies that are not only technologically sound but also culturally congruent, ensuring a more seamless integration into the Saudi healthcare system.

Privacy and security concerns

In examining privacy and security concerns within the context of EMR systems in Saudi Arabia, it is essential to consider the broader implications of technological trust and privacy perceptions. A study by Alqarni, Timko, and Rahman (2023) initially focused on the privacy and security implications of facial recognition technology (FRT). Despite its primary focus, the study's insights into privacy concerns and technological trust are notably applicable to EMR systems, highlighting the public's cautious stance towards privacy and security in digital healthcare solutions [28].

Further elucidating this point, research grounded in the "Diffusion of Innovation" theory, as proposed by Everett Rogers, provides a framework for understanding how innovations are adopted within a society. This theory suggests that the adoption of new technologies is influenced by factors such as perceived attributes of innovation, communication channels, time, and the social system. Applying this theory to the context of EMR systems, it becomes evident that privacy concerns significantly deter individuals' willingness to embrace smart government services, including EMR, due to apprehensions about data confidentiality and security [29].

The relevance of these concerns is further underscored by Alharbe's investigation in 2021 into the privacy and security of telehealth apps during the COVID-19 pandemic. The study emphasizes the critical need for transparent and responsible privacy-preserving practices in digital health technologies, a principle directly transferable to the implementation of EMR systems [30].

Moreover, the assessment by Bahaddad, Almarhabi, and Alghamdi (2022) on the acceptance of Bring Your Own Device (BYOD) programs sheds light on the intricate balance between user convenience and information security. Utilizing the unified theory of acceptance and use of technology (UTAUT) model, the research highlights the nuanced challenges of ensuring secure technology adoption in healthcare, reinforcing the necessity for robust security measures in EMR systems [31].

Policy and regulatory challenges

A study by Alshareef and Tunio (2022) examined the role of leadership in the adoption of blockchain technology in small and medium enterprises (SMEs) in Saudi Arabia. While focusing on blockchain, the study provides insights into policy and regulatory trends that can influence the adoption of innovative technologies, including EMR. It highlights the potential benefits and challenges that SMEs may face, which are relevant to healthcare institutions considering EMR adoption. The study underscores the need for attention to the complete mechanism of SMEs in promoting social and economic development, which can be paralleled in the healthcare sector [32].

Research on improving corporate stability through regulatory and financial reporting within the banking sector in Saudi Arabia provides insights into the challenges and developments in the financial sector. While focused on banking, this study offers perspectives on the impact of financial policy measures and regulatory changes, which are also relevant to the healthcare sector, particularly in the context of EMR adoption [33].

Al-Qahtani and Albakjaji (2023) assessed the legal framework governing foreign investment in Saudi Arabia, particularly in light of the National Investment Strategy. This paper's approach to identifying the legal framework and its effectiveness in achieving policy goals offers parallels to the regulatory challenges in EMR adoption. It emphasizes the need for a legal framework that aligns with Saudi Arabia's policies and goals, ensuring an effective and reliable regime for investors, which is also crucial for EMR system stakeholders [33].

Altobashi's (2019) investigation into corporate governance practices in Saudi Arabia-listed companies explored the emergence, development, and adoption of recent regulations. The study examined the different types of institutional pressures driving companies to implement governance practices, including challenges associated with adopting these regulations. This research provides insights into the policy challenges faced in adopting new regulations and practices, which are applicable to the healthcare sector's adoption of EMR systems [34].

Case studies and success stories

Salih et al. (2022) conducted a study on the critical success factors (CSFs) for enterprise resource planning (ERP) system implementation in the Saudi Arabian food industry. This research is relevant to EMR adoption as it highlights the importance of user acceptance, project management, and top management support in the successful implementation of ERP systems. These factors are also critical in EMR adoption, emphasizing the need for effective management and user training [35].

Another study by Salih et al. (2022) examined the influence of top management and vendor support as critical success factors in the post-implementation stage of ERP systems in SMEs in Saudi Arabia. The findings suggest that continuous vendor support and effective communication between departments significantly impact the success of ERP systems. This study provides insights into the importance of ongoing support and communication in the successful adoption of EMR systems [36].

Andejany et al. conducted research on the critical success factors of the Tawakkalna application in Saudi Arabia during the COVID-19 pandemic. The study focused on the effectiveness of the app in managing health status checks and proposed solutions to improve the process. This case study offers valuable lessons for EMR implementation, particularly in terms of user acceptance and efficiency in healthcare operations [37].

Alharbe's research aimed to develop an EMR implementation framework for public hospitals in Saudi Arabia. The study addressed the barriers to and facilitators for implementing EMRs within healthcare organizations, considering cultural, resource-related, and technological factors. This research provides a comprehensive framework that can guide successful EMR implementation in Saudi public hospitals [30].

Collectively, these studies provide a broader perspective on the successful implementation strategies for EMR systems in Saudi Arabia, highlighting the importance of user acceptance, management support, vendor collaboration, and tailored implementation frameworks.

A spectrum of challenges and successes marks the journey of EMR adoption in Saudi Arabia. Addressing technical, financial, human, cultural, and policy-related obstacles while leveraging the lessons from successful implementations is essential for advancing the healthcare delivery system in line with Saudi Arabia's Vision 2030 objectives.

Emerging trends and future directions in EMR adoption

As we delve deeper into the literature surrounding the adoption of EMR in Saudi Arabia, it becomes evident that while substantial progress has been made, there remain significant gaps and emerging trends that merit further exploration. These areas not only offer fertile ground for future research but also promise to significantly contribute to the evolving narrative of healthcare digitalization, particularly within the dynamic context of Saudi Arabia's healthcare sector.

A pivotal area for future investigation is the nuanced interplay between cultural norms and organizational structures in Saudi Arabia and their impact on EMR adoption. Understanding these intricacies is essential for devising EMR implementation strategies that resonate with the cultural fabric and organizational realities of the Saudi healthcare system. Moreover, the integration of artificial intelligence (AI) and big data analytics into healthcare systems presents an important frontier for research. Investigating the potential of AI to enhance EMR systems through predictive analytics for patient care and operational efficiencies is a critical area of inquiry that could yield transformative insights for EMR adoption in Saudi Arabia.

Privacy and security concerns have emerged as paramount considerations in our review, underscoring the need for future research to assess and innovate effective privacy-preserving methods within EMR systems. This could involve exploring the adoption of blockchain technology, advanced encryption methods, and privacy-by-design approaches to ensure the protection of patient data.

The impact of evolving healthcare policies and regulations, particularly in response to technological advancements and global health challenges, on EMR adoption and integration is another crucial area for investigation. Future studies should aim to analyze how recent regulatory changes influence EMR implementation efforts and identify potential areas for policy enhancement to facilitate smoother adoption processes.

The patient's role in the EMR adoption process represents a relatively underexplored dimension. Future research could provide valuable insights by focusing on patient attitudes toward EMR, their concerns regarding privacy and data security, and the potential of EMR systems to foster patient engagement and self-management of health.

Furthermore, the accelerated adoption of telemedicine services, propelled by the COVID-19 pandemic, highlights the importance of examining how telemedicine can be effectively integrated with EMR systems. Such research could elucidate ways to enhance healthcare delivery, patient outcomes, and system efficiency in Saudi Arabia through seamless EMR and telemedicine integration.

Exploring these evolving trends and uncharted territories in the existing research can profoundly enhance our grasp of the hurdles and prospects of adopting EMR within the Saudi Arabian healthcare landscape. This quest is more than an academic exercise; it's about weaving a richer narrative around the digitization of healthcare. It fosters a deeper dialogue among scholars and practitioners about how technology can transform patient care and aligns with the broader objectives of Saudi Arabia's Vision 2030 for a more innovative and efficient healthcare system.

## Conclusions

This review has discussed the complexities surrounding the adoption of EMR in the context of Saudi Arabia's ambitious Vision 2030 healthcare objectives. We have uncovered a spectrum of challenges ranging from technical issues affecting system reliability and trust to financial hurdles that hinder resource allocation and human factors that impact the willingness of healthcare professionals to embrace EMR systems. Additionally, the nuances of cultural and organizational resistance, alongside concerns about privacy and security and the intricacies of policy and regulatory frameworks, have emerged as critical elements influencing EMR adoption.

Our findings underscore the necessity for a holistic approach that encompasses thoughtful strategic planning, the development of intuitive and user-centric EMR systems, extensive training programs, and stringent privacy safeguards. The lessons drawn from success stories and practical case studies illuminate the path forward, highlighting the indispensable roles of visionary leadership, collaborative stakeholder engagement, and flexible implementation strategies. For Saudi Arabia to effectively integrate EMR systems into its healthcare infrastructure, it is imperative to confront and overcome these challenges head-on. This review not only sheds light on the obstacles at hand but also serves as a beacon for policymakers, healthcare practitioners, and industry stakeholders, guiding them toward informed decisions and actions that will elevate the quality and efficiency of healthcare services. As we move forward, surmounting these barriers will be crucial in realizing the transformative potential of EMR systems, thereby contributing significantly to the realization of Vision 2030's healthcare aspirations.
